# Editorial: Age as an effect modifier of nutrition and health

**DOI:** 10.3389/fragi.2023.1249377

**Published:** 2023-07-12

**Authors:** Jirayu Tanprasertsuk, Aron Keith Barbey, Elizabeth J. Johnson

**Affiliations:** ^1^ NomNomNow Inc., Nashville, TN, United States; ^2^ Beckman Institute for Advanced Science and Technology, University of Illinois at Urbana-Champaign, Champaign, IL, United States; ^3^ Friedman School of Nutrition Science and Policy, Tufts University, Medford, MA, United States

**Keywords:** older adult, nutrition, diet, epidemiology, health, aging

As we progress through the stages of life, our nutritional needs undergo changes influenced by the aging process and its impact on our health. Given the global increase in the elderly population and the heightened risk of chronic diseases and functional decline among older adults, it is vital to identify optimized diets for this demographic. This Research Topic explores recent studies that shed light on the nutritional challenges faced by older adults, offering practical examples of dietary and lifestyle modifications that inform comprehensive interventions and policies.

Seid and Babble article brings attention to the demographic shift and rising life expectancy in Africa, resulting in a growing population of older adults. The authors underscore the severe health consequences and reduced quality of life associated with malnutrition among the elderly. Through a systematic review and meta-analysis, their research reveals that malnutrition is a significant issue across various African countries and regions, as measured by body mass index or the Mini Nutritional Assessment. Additionally, Chareh et al. study addresses the poorly understood connection between inflammation and appetite, which may contribute to undernutrition in older adults. Their findings indicate that heightened inflammation levels, as indicated by elevated CRP and IL-6 levels, are linked to reduced appetite in this population. This study validates the need for further research on managing appetite dysregulation and undernourishment among the elderly.

Observational studies provide a substantial foundation for designing dietary and lifestyle interventions. Lizzo et al. conducted a short-term randomized controlled trial to investigate the potential benefits of glycine and N-acetylcysteine supplementation on antioxidant defense and oxidative stress in healthy older adults. The results demonstrated that both supplements led to improvements in glutathione redox status and a reduction in oxidative damage markers compared to the placebo group. These findings suggest the potential of these supplements in mitigating age-related oxidative stress and improving overall health. Similarly, Bischoff-Ferrari et al. conducted a long-term trial to examine whether a combination of vitamin D supplementation, omega-3 fatty acids supplementation, and a simple home exercise program could reduce the incidence of cancer in older adults. They found that the combined intervention resulted in a significant reduction in the incidence of total cancer among active older adults compared to the placebo group. However, when each intervention was assessed individually, no significant reduction in cancer risk was observed. This study highlights the potential benefits of a multi-component approach in cancer prevention strategies.

The exemplary works presented in this Research Topic provide valuable insights into addressing the nutritional challenges faced by aging populations. However, further high-quality research is still needed, including improved metrics and data collection, to inform nutrition and food policies specifically tailored to the aging population. Such efforts will support the advancement of nutrition science and provide evidence-based recommendations for healthy diets throughout each stage of life.

